# Neurohormones in the Pathophysiology of Vasovagal Syncope in Adults

**DOI:** 10.3389/fcvm.2020.00076

**Published:** 2020-05-06

**Authors:** David G. Benditt, J. Gert van Dijk, Darshan Krishnappa, Wayne O. Adkisson, Scott Sakaguchi

**Affiliations:** ^1^Cardiovascular Division, Department of Medicine, Cardiac Arrhythmia and Syncope Center, University of Minnesota Medical School, Minneapolis, MN, United States; ^2^Department of Neurology, Leiden University Medical Center, Leiden, Netherlands

**Keywords:** vasovagal syncope, neurohormone, neuroendocrine, catecholamines, tilt-table testing

## Abstract

Vasovagal syncope (VVS) is the most common cause of syncope across all age groups. Nonetheless, despite its clinical importance and considerable research effort over many years, the pathophysiology of VVS remains incompletely understood. In this regard, numerous studies have been undertaken in an attempt to improve insight into the evolution of VVS episodes and many of these studies have examined neurohormonal changes that occur during the progression of VVS events primarily using the head-up tilt table testing model. In this regard, the most consistent finding is a marked increase in epinephrine (Epi) spillover into the circulation beginning at an early stage as VVS evolves. Reported alterations of circulating norepinephrine (NE), on the other hand, have been more variable. Plasma concentrations of other vasoactive agents have been reported to exhibit more variable changes during a VVS event, and for the most part change somewhat later, but in some instances the changes are quite marked. The neurohormones that have drawn the most attention include arginine vasopressin [AVP], adrenomedullin, to a lesser extent brain and atrial natriuretic peptides (BNP, ANP), opioids, endothelin-1 (ET-1) and serotonin. However, whether some or all of these diverse agents contribute directly to VVS pathophysiology or are principally a compensatory response to an evolving hemodynamic crisis is as yet uncertain. The goal of this communication is to summarize key reported neurohumoral findings in VVS, and endeavor to ascertain how they may contribute to observed hemodynamic alterations during VVS.

## Introduction

Vasovagal syncope (VVS) is the most frequently encountered form of reflex syncope, and is the most common cause of syncope across all age groups (1, 2). Although the clinical importance of VVS has been widely acknowledged for more than a century, and despite considerable investigational effort, its pathophysiology remains incompletely understood. In this regard, several reports have examined neurohumoral changes accompanying VVS (primarily VVS induced during head-up tilt table testing) in an attempt to gain insight into the basis of the hemodynamic alterations associated with VVS episodes. For the most part, studies have focused on changes in circulating catecholamines prior to and during VVS. However, a number of other neurohormones have also been the subject of study, including vasopressin, endothelin-1, adrenomedullin, brain and atrial natriuretic peptide (BNP, ANP) and serotonin. In this report we aim to summarize the key reported neurohumoral changes that have been observed during VVS, and endeavor to relate them to its pathophysiology.

## Overview of VVS Pathophysiology

VVS pathophysiology remains a subject of active study with many residual unknowns (1–4). From a clinical perspective it is understood that susceptibility to VVS varies both among and within individuals over time. In this regard, multiple different VVS triggers (e.g., emotional upset, pain, venipuncture, and volume depletion), may initiate an event. However, even though it is believed that all humans (but not other species) are believed to be appropriately “wired” to permit VVS, certain individuals seem to be more susceptible to VVS triggers than are others. Additionally, VVS induction is inconsistent. The same stimuli do not always initiate episodes, even in individuals in whom VVS has previously occurred under similar circumstances.

Genetic predisposition may account in part for inter-individual variability of VVS susceptibility, but genetics cannot not readily address temporal variations of susceptibility within individuals. On the other hand, as yet poorly understood variations of both neural and neuroendocrine responses to triggers may play a role in determining whether a VVS event is initiated. With respect to neurohormone contributions to VVS (the subject of this communication), the relative magnitude of release of various endogenous vasoactive substances and their interaction at a given time, might be expected to vary, and thereby contribute to variation in the likelihood of VVS occurring at any point in time or in response to a particular “trigger.” In this regard, a number of neuroendocrine changes have been identified as being associated with VVS events; however, whether they have a causation role, or are predominantly bystander (possibly even compensatory given ongoing hemodynamic changes) effects remain subjects of ongoing study.

Certain clinical observations are relevant to the understanding of VVS pathophysiology. Most importantly, VVS events tend to occur while the affected individual is in an upright position, and are extremely rare when patients are supine. Thus, it is reasonable to assume that gravity contributes, and that venous pooling is a crucial element of the pathophysiological process; in large measure the ability of head-up tilt (HUT) table testing to trigger VVS relies on this aspect of VVS physiology. However, whether the pathophysiology of VVS induced by head-up tilt is representative of VVS associated with other triggers (e.g., pain, emotional upset, dehydration) is unknown.

The pooling of blood below the diaphragm (i.e., in the thighs, buttocks, splanchnic bed, and possibly the pelvic organs) during upright posture inevitably diminishes venous return to the heart. The consequence is decreased right atrial volume, reduced pulmonary artery blood flow, and ultimately diminished cardiac output.

Reduction in venous return and an inappropriate reduction of cardiac output (CO), would be expected to trigger an increase in efferent sympathetic neural tone in an attempt to increase heart rate (a compensatory means to restore CO) and vascular tone in order to maintain both organ perfusion and systemic blood pressure (3, 5–8). The main drivers of these compensatory responses are primarily afferent signals from cardiac and vascular baroreceptors to central nervous system cardiovascular control centers in the mid-brain. Attempts have been made to discern which of the pressure/stretch receptors (i.e., atrial, arterial) are the more important for initiating VVS, but this has proved difficult. In any case, the afferent signals are directed to the nucleus tractus solitarii in the medulla oblongata, where integration with other incoming inputs takes place (3). Subsequently, in an evolving VVS there is a sequence of neural and hormonal changes that are reasonably interpreted at least initially as a compensatory attempt to prevent hypotension. Not infrequently, the compensation is effective in stabilizing the circulation, and syncope does not occur. Nevertheless, patients may experience certain “warning” symptoms that spontaneously resolve including palpitations, abnormal temperature sensations and gastrointestinal upset. However, if compensation fails, then the VVS episode progresses to include diminished cardiac output with hypotension.

Reports vary regarding the changes in sympathetic nerve activity that occur VVS in susceptible patients (4). Some studies suggest that diminution of sympathetic tone as assessed by microneurographic sympathetic nerve activation (MSNA) recordings cause loss of venous and arterial tone (i.e., vasodepression) resulting in progressive reduction of venous return and arterial pressure (3). On the other hand, others have noted preservation of MSNA throughout an evolving VVS has also been observed until the development of syncope. The apparent discordance in MSNA observations during induced VVS requires further study. In this regard a few possible explanations merit investigation, including: (1) unknown factors, other than sympathetic nerve activity, determine vascular resistance status, and (2) MSNA recordings, by virtue of being able to assess a limited number of accessible nerves, may not be recording those nerves most pertinent to the evolving hypotension, (3) despite maintenance of MSNA, hypotension may occur if NE release is impaired at the synapse or NE re-uptake is enhanced.

The late phase of VVS is associated with an increase in parasympathetic tone. The latter, tends to result in a marked or relative cardioinhibition, thereby undermining any attempt to provide a compensatory chronotropic response in the face of evolving hypotension.

## Specific Neurohumoral Alterations Accompanying VVS

A wide range of neurohumoral changes have been reported to be associated with evolving VVS episodes. Most findings have been observed using head-up tilt (HUT) triggered VVS, while a few have been obtained during VVS induced by lower body negative pressure (LBNP).

Alterations of circulating catecholamines during evolving VVS have been the subject of study since the mid-1960s, with some difficult-to-explain differences being reported. The first observations are usually credited to Chosy and Graham (9) who noted higher urinary epinephrine (Epi) levels in blood donors who went on to faint than in others who did not faint. Subsequently, others provided more detailed evaluation of catecholamine changes in VVS, along with assessment of a variety of other neurohumoral agents to be discussed later. The principal observations are summarized here.

### Catecholamines

The possibility that circulating catecholamines play a role in VVS pathophysiology has been the subject of interest for more than 30 years (3, 6–20). The most consistent finding has been a relatively early increase in circulating epinephrine (Epi) during head-up posture prior to the faint. The basis for this Epi increase is as yet unexplained.

In most instances, the Epi concentration at time of syncope was higher than baseline and much higher than observed after comparable periods of head-up posture in non-fainters. However, in a few reports the Epi levels were not significantly increased (13, 15). Reasons for the differing outcomes remain unclear, although in the report by Vanderheyden et al. (15), the mean value of Epi did increase but the standard deviations in a relatively small population was large and may have obscured any statistically significant change.

In terms of key studies examining catecholamines during induction of VVS, Fitzpatrick et al. (10) observed a substantial increase of circulating epinephrine (Epi) concentrations in association with an imminent faint in 7 tilt-table induced fainters, but not in 2 control subjects and 2 other individuals who had a fainting history, but in whom the HUT did not trigger an event. Further, Epi concentrations were somewhat higher in fainters at baseline and at 10 min of tilt when hemodynamics were stable, However, in close temporal relation to the faint, epinephrine concentration was substantially higher in HUT-positive fainters than in HUT-negative non-fainters. Norepinephrine (NE) also increased during HUT but did not differ significantly in the 2 groups for the duration of the test. These same investigators also identified a marked increase in circulating arginine vasopressin (AVP) and pancreatic polypeptide (PPP) during upright posture in fainters compared to controls, topics that will be addressed more fully later.

In the report by Sra et al. (11), a somewhat larger population was studied (30 individuals; 19 VVS subjects and 11 controls). These researchers also observed that NE increments with head-up posture were comparable in VVS patients and controls, but that Epi increments were much greater in the VVS subset (~5-fold increase) compared to minimal change in control subjects. The authors concluded that VVS is associated with diminished neuronal sympathetic NE release, and enhanced adreno-medullary activity.

**Figure 1:**
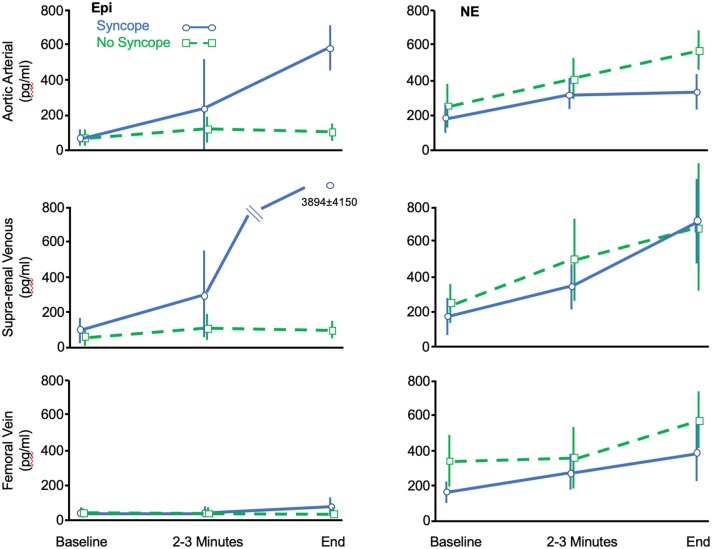
Data adapted from Ermis et al. (16) showing change of circulating Epi and NE concentrations (ordinate: pg/ml) measured at various anatomic sites during the course of HUT-induced syncope (Blue) or HUT without syncope (Green). See text for details.

The most detailed early evaluation of hemodynamic and neurohumoral changes in evolving VVS was provided by Jardine et al. (14) using prolonged head-up tilt at 60 degrees. Norepinephrine (NE) and epinephrine (Epi) increased early during upright posture to a greater extent in fainters than controls. However, in proximity to syncope the Epi level continued to rise while NE fell back to control values. Ermis et al. (16, 17) found that the NE values continued to rise throughout the HUT but were similar in both fainters and controls, with no fallback in the fainter group at the time of symptoms. However, consistent with most of the reports summarized above, Epi continued to rise, and reached values 6 to 15 times baseline depending on the site from which the blood was drawn (i.e., femoral vein, aorta, renal vein-vena caval junction) (Figure 1). In essence, near the time of head-up tilt induced faint, all reports agree that the Epi/NE ratios were much higher in fainters than was the case at baseline in the same individuals or in comparable control subjects.

Using lower body negative pressure method, Lenders et al. (18) concluded that there was larger synaptic norepinephrine “spillover” (i.e., NE which reaches the circulation) in non-fainters than fainters. This observation implies several possibilities in non-fainters compared to fainters: (1) greater NE production, (2) more NE being released at the synapse, or (3) less NE being re-captured by the synaptic re-uptake process. The latter observation suggested that perhaps there was a defect of neural NE production in fainters or an unexpected augmentation of NE reuptake in the synapse.

In regard to the NE neural production issue, Vaddadi et al. reported reduced tyrosine hydroxylase activity, an important rate-limiting step in NE synthesis in VVS subjects (20). The latter occurred predominantly in patients who presented with a low blood pressure phenotype of VVS as initially described by Mathias et al. (21). The same investigators also observed higher NE transporter (i.e., increased re-uptake capability within the synapse) in the normotensive blood pressure phenotype of VVS patients. Both of these functional sympathetic nerve defects would be expected to reduce NE spillover into the circulation, and thereby be consistent with the diminished NE levels summarized above. On the other hand, if these NE production and/or NE re-uptake issues are confirmed to be prevalent in VVS susceptible subjects, it raises the issue of why VVS only occurs intermittently and not reproducibly. Potentially, NE production/re-uptake may vary with time, a possibility that would be very difficult to study.

The apparent disturbance of NE release and/or uptake was further assessed by Ermis et al. (16, 17), who also noted that while circulating NE rose throughout HUT, the increments with upright posture in fainters were similar to that in controls and much less than might have been expected given the hemodynamic crisis. However, they also observed that to some extent NE release at the level of the renal/adrenals may provide some element of compensation (Figure 1). Specifically, Ermis et al. (16) reported that there was near doubling of NE concentrations near the time of syncope between femoral venous sites and supra-renal sites in fainters but not in non-fainters (714 ± 476 pg /ml vs. 385 ± 166 pg/ml, *P* < 0.05). The authors speculated that this latter NE increment was derived from the kidneys or adrenal gland and may have provided some compensation for failure of synaptic NE contribution to maintain hemodynamic stability. It also suggests that the drivers for NE release may differ at the neural synapse vs. adrenal/renal sites; if that were the case, perhaps any postulated issues with NE production/re-uptake noted earlier, may not apply to the same extent in the adrenal glands or kidney. At present, the basis for this seeming difference between neural and organ NE overflow is unknown.

**Figure 2:**
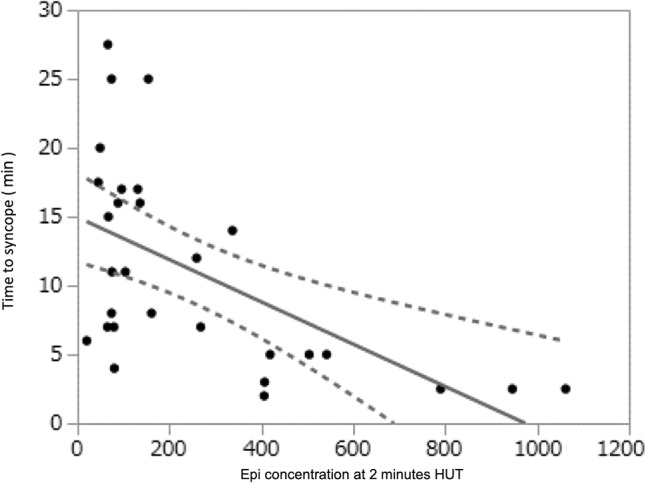
Data derived from Kohno et al. (19) showing that the time to syncope during HUT was shorter (Time in Minutes on ordinate) as the Epi concentration increased (abscissa, pg/ml).

More recently, the relationship between tilt-induced increase of circulating catecholamines (particularly Epi) and time to HUT-induced VVS (i.e., the latter being used as a surrogate measure of “susceptibility” to VVS) has been introduced for use in the clinical laboratory. Kohno et al. (19) observed a significant correlation between higher baseline and 2-min plasma Epi level and shorter time to syncope (baseline: *R*-Squared = 0.12, *P* = 0.048, and 2 min : R-squared = 0.33, *P* = 0.001) (Figure 2). Similarly, there was a significant correlation between greater Epi/NE ratio at 2 min and shorter time to syncope (R-squared=-0.49, *P* = 0.007). Finally, a greater increase of Epi levels from baseline to 2 min of HUT (i.e., difference 2-min Epi minus baseline Epi) was associated with a shorter time to syncope (*R* = -0.58, *P* = 0.001). On the other hand, with respect to NE alone, neither 2-min HUT levels nor change from baseline values correlated with time to syncope.

In an even more recent study of a large group of VVS susceptible individuals, Torabi et al. (22) reported findings very similar to those of Kohno et al. (19).

In summary, VVS triggered by head-up posture appears to be associated with marked increases in circulating catecholamines even prior to hypotension; circulating epinephrine levels seem to increase particularly dramatically. However, whether these changes are causal remains uncertain. An epinephrine (Epi) relation to VVS susceptibility seems likely given the consistency of the finding of increased Epi levels across many studies. However, if Epi or NE changes contribute directly to VVS pathophysiology, the manner in which they participate is as yet uncertain. One initial concept was that Epi/NE enhance ventricular force of left ventricular contraction and thereby stimulate myocardial wall mechanoreceptor afferent signaling, with a subsequent reflex lowering of heart rate and blood pressure. However, this mechanism is not widely held given the observation of VVS after heart transplantation. Potentially, other non-cardiac arterial receptors may be operating in parallel thus maintaining a modified version of the basic theory. In any case, while at best only an indirect argument in favor, the physiologic actions of a greater Epi/NE ratio is appropriate to lead to clinical features consistent with VVS (e.g., vascular dilatation in some beds with constriction in others such as the skin). Nevertheless, this interpretation of the role of catecholamines has not been without controversy, especially given the failure of adrenergic blockers to show a universal clear preventative benefit in VVS susceptible patients (23).

### Vasopressin

Arginine vasopressin (AVP), is an endogenous nonapeptide hormone synthesized in the hypothalamus and subsequently transported via neuronal axons to the posterior pituitary gland where it is able to access the circulation (24, 25). Release into the circulation from pituitary capillaries is facilitated by their lack of blood-brain barrier. At usual circulating concentrations, AVP's action is primarily antidiuretic (hence its other common name: antidiuretic hormone). However, at higher than physiologic levels such as might be expected during a hypotensive crisis, AVP is a potent vasoconstrictor (26).

In health, AVP is not deemed important for maintaining cardiovascular homeostasis. However, in a crisis (e.g., severe hemorrhage), endogenous circulating AVP may increase sufficiently to provide compensatory benefit. AVP interacts with 3 receptors (V1: predominantly vascular effects, V2:which mainly act to retain water, and V3: which are mainly in neurons, particularly the adenohypophysis leading to ACTH release) (24, 25). On the other hand, acting centrally, AVP enhances baroreceptor sensitivity. The expected result of the latter action would be increased vagotonia and lower sympathetic tone relative to the level of blood pressure (this may be important in VVS as discussed later).

Increased levels of AVP in association with VVS were first reported by Riegger and Wagner (27) and Fitzpatrick et al. (10), and again somewhat later by others including Jardine et al. (14) and Theopistou et al. (28), Rash et al. (29) and Nilsson et al. (30). The report by Fitzpatrick et al. (10), although encompassing a small patient population, indicated that baseline AVP was higher in fainters and that remained the case at 10 min of HUT when hemodynamics were still stable. With syncope, AVP was markedly increased. Jardine et al. (14) and Theopistou et al. (28) similarly noted that AVP increased during HUT in fainters, although only modestly or not at all in the early stages of the procedure when hemodynamics were stable. However, at the time of syncope, AVP was 20-fold higher than baseline (28). On the other hand, Rash et al. (29) did not observe any evident baseline AVP differences among 3 groups of patients (VVS, epilepsy and controls) but they did not provide measures during or in proximity to syncope events. Finally, Nilsson et al. (30) found AVP to be higher in patients who fainted spontaneously during HUT compared to those who fainted after nitroglycerine stimulation. The pathophysiologic implication of this latter set of observations is not immediately evident.

In summary, Fitzpatrick et al. (10), Jardine et al. (14) and Torabi et al. (22) observed moderately increased AVP values early in HUT in VVS prone patients suggesting a potential marker of susceptibility, but others did not detect baseline differences (28, 29, 31). Nonetheless, all confirmed that AVP increased abruptly in close proximity to the faint itself. Thus, while one cannot yet be certain, it appears that the elevated AVP levels are more likely reactive to evolving hypotension rather than a marker of susceptibility. On the other hand, as discussed below, AVP may ultimately contribute as an additional provocateur, not so much as a compensatory actor.

The role of AVP in blood pressure control is not fully settled (24, 32), and assessing its actions in a setting in which catecholamines are also increased is particularly problematic. Most investigators agree that short-term blood pressure control is primarily neurally-mediated rather than humoral. AVP, as a vasoconstrictor, may only become important during a hemodynamic crisis (26). In regard to VVS, and based on the preponderance of observations summarized above, it seems that a substantial AVP rise occurs primarily in response to a serious hypotensive crisis (i.e., the final phase of the VVS event). The latter is consistent with the findings of Goldsmith et al. (26) showing that in humans AVP release to levels that may have vasoconstrictive capability (i.e., potentially compensatory in the setting of VVS-induced marked hypotension) is only triggered by very severe drops in systemic pressure. Nonetheless, whatever the trigger for marked AVP release near the end of a VVS event, it is inadequate to reverse the progressive and often dramatic blood pressure fall in patients destined to faint.

Why AVP fails to reverse the hypotensive trend even when finally released in substantial concentrations is not immediately obvious. However, the explanation may lie in the net outcome of two potentially contrary AVP actions: (1) a direct AVP constriction effect, and (2) reflex effects initiated by AVP-induced increase in central venous pressure that triggers low pressure atrial mechano-receptors (see earlier). In brief, Aylward et al. (24) noted that exogenous AVP infusion to levels known to have an antidiuretic effect had little hemodynamic impact in healthy men. Further reflex changes induced by neck pressure, neck suction, or lower body negative pressure (LNBP) were unaffected. However, at higher AVP doses, there was unexpected forearm vasodilation; this being a vascular bed that seems to be strongly influenced by low pressure atrial receptors (24). Further, reflex vasodilation triggered by release of LBNP was augmented in the presence of AVP. Thus, under certain conditions in humans, the net effect (i.e., constriction vs. baroreceptor anti-sympathetic action) of AVP may facilitate reflex vasodilation. If that occurs in an evolving VVS scenario in which (like LNBP) there is diminished venous return, AVP may exacerbate the blood pressure (BP) problem. However, as BP plummets, the AVP reflex anti-sympathetic action may diminish and peripheral resistance may then have an opportunity to increase and restore hemodynamic stability.

In conclusion, multiple studies are consistent in reporting marked increase of AVP in temporal proximity to a VVS event. However, resting AVP levels do not appear to be a marker of VVS susceptibility, as they are not consistently different in VVS susceptible patients and controls. Consequently, the terminal-VVS AVP rise in fainters is most likely a reaction to serious systemic hypotension. However, and paradoxically perhaps, the marked AVP rise in the setting of reflex hypotension may exacerbate the drop in pressure via an effect on baroreceptor sensitivity. The latter are active in the basal state, providing vagal tonus and sympathetic inhibition. AVP may enhance these effects by increasing central venous pressure. In other non-reflex hypotensive circumstances (e.g., severe hemorrhage) in which a central venous pressure fall cannot be readily reversed by AVP, the net action of an increased AVP level may be of compensatory value. Clearly, further study of AVP action in VVS is needed.

### Endothelin-1

Endolthelin-1 (ET-1) is a 21 amino acid peptide vasoconstrictor and pro-inflammatory agent derived from a 39 amino acid precursor through an endothelin converting enzyme (33). ET-1 is one of 3 isoforms (ET-1, ET-2, ET-3), but is the most important from a cardiovascular perspective.

ET-1 which is primarily derived from endothelial cells, may be viewed as balancing the effects of nitric oxide (NO). Its release is also inhibited by NO, atrial natriuretic peptide (ANP), and by prostacyclin. Conversely, ET-1 release may be promoted by humoral factors including AVP, angiotensin II, as well as physical factors such as vascular shearing forces. Under pathological conditions, ET-1 may be produced by other cells including vascular smooth muscle cells and cardiac myocytes. ET-1 effects act through 2 receptors (ET_A_, ET_B_), with the net effect (constriction vs. NO release by ET_B_) being determined by the location and balance between receptor sites (33).

Several studies have examined ET-1 in VVS patients. Rash et al. (29) found no difference in baseline ET-1 levels among syncope, seizure and control subjects. Magerkuth et al. (34) found elevated ET-1 in tilt-test positive patients both on the day of the test and at other times suggesting that ET-1 may be a marker for susceptibility. However, baseline differences were not found by either White et al. (35) or Kaufmann et al. (36). The former noted that orthostatic stress increased ET-1 in controls, but not in VVS positive patients, while Kaufmann et al. found ET-1 to rise similarly with upright tilt in VVS patients and in controls, but not at all in autonomic failure patients. They concluded, that during orthostatic stress the ET-1 increase is mediated by ET-1 released from the neurohypophysis rather than the vascular periphery. In this regard, Fedorowski et al. (37) came to an analogous conclusion suggesting that increased ET-1 is more consistent with neurogenic non-VVS orthostatic cause of syncope, while low ET-1 is a marker of VVS. While such a conclusion requires more evidence, these three reports are consistent (34–37); in essence, lower ET-1 levels were associated with greater likelihood of initiating VVS. Presumably, diminished ET-1 induced vasoconstriction may enhance VVS susceptibility.

### Adrenomedullin

Adrenomedullin (ADM) is a 52-amino acid multifunctional peptide vasodilator (acting via NO) and natriuretic agent that was first isolated from pheochromocytoma tissue in the early 1990s (38). It is widely accepted that measurement of the mid-regional fragment (MR-proADM) is a useful surrogate for ADM itself. High ADM levels have been associated with POTS in children (39).

The role of ADM in VVS has been the subject of several reports but currently its impact remains unclear. Plasek et al. (40) did not observe a significant difference in ADM levels in a study comparing of 14 HUT-positive patients with a cohort of 14 HUT-negative control group. Gajek et al. (41) observed ADM to rise substantially in patients who developed VVS during the passive phase of HUT (but not if nitroglycerin-induced) On the other hand, Hamrefors et al. (42) observed that in patients >40 years of age, lower supine MR-pro-ADM predicted asystolic VVS on HUT, although orthostatic levels of the biomarker were not assessed. They also noted a tendency toward a relationship between ADM levels and tendency to cardioinhibitory forms of VVS; in brief, lower ADM levels were associated with greater likelihood of cardioinhibition. The highest ADM levels were noted in HUT-negative subjects. More recently, Torabi et al. (22) found that higher baseline MR-pro-ADM during HUT was associated with longer time to syncope. Thus, for unclear reasons higher ADM levels appears to be “protective,” whereas intuitively the opposite would be expected. In any case, in the largest study to date examining baseline ADM levels, Fedorowski et al. (37) in multivariable analysis did not find it to be an independent marker of VVS susceptibility.

### Atrial (ANP) and Brain (BNP) Natriuretic Peptide

The potential contributions of brain and atrial natriuretic peptides (BNP and ANP) on VVS induced by HUT were first examined by Jardine et al. (14) in VVS patients and control subjects. In that study, BNP did not differ in the two patient groups, and did not change substantially throughout HUT and recovery; consequently, BNP has for the most part not been deemed relevant to VVS pathophysiology. However, it has been suggested that BNP may be a useful diagnostic marker in for cardiac syncope, but that issue will not be discussed further here.

In regard to ANP, Jardine et al. (14) found that while ANP levels are higher in syncope-prone patients compared to controls, they were not appreciably altered by whether syncope occurred or not. In fact, ANP levels tended to decline in both groups throughout the tilt procedure. Subsequently, Fedorowski et al. (37) examined mid-regional fragments of pro-atrial natriuretic peptide (MR-proANP); findings revealed that ANP levels tended to be lowest in patients prone to tilt-induced fainting and highest in non-fainters. Consequently, the role played by ANP, if any is unclear and difficult to explain. Specifically, while increased ANP levels in isolation may largely reflect atrial stretch status, the latter may be affected by other influences occurring at the same time analogous to the complex effects of AVP discussed earlier.

### Galanin

Galanin is a neuropeptide that is widely distributed in the central and peripheral nervous systems (43). Galanin interacts with both sympathetic and vagal systems as well as with neurotransmitters, such as serotonin. In animals, galanin can lower blood pressure and attenuate vagally-induced slowing of the heart rate. In humans, the administration of galanin depresses basal norepinephrine and norepinephrine responses to both assumption of upright posture and insulin-induced hypoglycemia.

Bondanelli et al. (44) examined plasma galanin changes during HUT in healthy subjects and patients with recurrent VVS. In healthy subjects, galanin did not change during HUT. However, in VVS patients with a negative response to tilting (no syncope), galanin significantly (*P* < 0.001) increased and correlated positively with the increases in blood pressure (BP) and heart rate (HR). In control patients with a positive HUT response (i.e., false positive), galanin did not change either before the loss of consciousness or during syncope. Thus, circulating galanin levels progressively increased during a negative HUT in patients with a history of VVS, whereas they remain unchanged in healthy subjects. Moreover, in the patients with tilting-induced syncope galanin does not change either before or during loss of consciousness. Potentially increased Galanin acted to diminish VVS susceptibility.

Plasek et al. (40) obtained slightly different results. These authors observed an increase in plasma galanin for VVS patients during HUT, regardless of whether they developed syncope or not during the test. Conversely, orthostatic stress was associated with galanin decrease in controls. Thus, galanin might be useful as a marker for VVS susceptibility, but further evaluation is needed.

### Pancreatic Polypeptide

Pancreatic polypeptide is a 36-amino acid peptide secreted from the pancreas. Its primary action is in the gastrointestinal system where its release is known in part to be mediated by vagal activity. It helps regulate pancreatic hormone release and may have satiety actions.

In regard to VVS, pancreatic polypeptide has been reported to be increased in conjunction with the faint (10, 14). Jardine et al. (14) observed a statistically significant greater increase in pancreatic polypeptide during HUT compared to controls in whom there was no change from baseline. Fitzpatrick et al. (10) observed no difference between HUT positive fainters and HUT negative controls during the tilt test procedure. However, pancreatic polypeptide increased markedly during HUT recovery in fainters but was unaffected in control subjects.

In summary, it is unlikely that pancreatic polypeptide has a mechanistic action in VVS, other than as a marker of increased vagal activity in temporal proximity to the faint. Perhaps an elevated measurement after recovery may be a marker of a recent VVS event, but further evaluation is needed before such an application can be recommended.

### Others (Cyclic AMP, Opioids, Plasma Renin Activity, Angiotensin II, and Serotonin)

#### Cyclic AMP

Cyclic AMP (cAMP) is an intracellular second messenger, that transduces the effects of a number of hormones (e.g., epinephrine) that cannot enter cells themselves. Abe et al. (12) examined changes in cyclic-AMP during HUT-induced VVS. However, the use of isoproterenol in the HUT protocol somewhat complicates interpretation of the results, but HUT with isoproterenol was associated with a c-AMP increase with movement to upright posture and a further increase if syncope was induced. However, in terms of magnitude, the c-AMP increase of ~16% was less in percentage terms than was the case for NE, being ~44%. Beta-blockers, particularly propranolol, blocked both c-AMP changes and syncope induction in this report.

Mitro et al. (45) also examined c-AMP in VVS although they did not undertake a c-AMP measurement to determine if a premonitory change was evolving at the crucial point during upright posture when hemodynamics were stable to determine if a premonitory change was evolving. In their report, 61 syncope patients (age 35 ± 15 years) underwent a passive HUT. Blood samples for NE, Epi, and dopamine were obtained at baseline supine, at 5 min of HUT and at syncope or end HUT (45 min). cAMP values were obtained at baseline and at the end HUT. HUT was positive for VVS in 33 and negative for VVS in 28 patients. There were no significant neurohumoral baseline difference, but while NE, Epi and dopamine were higher with HUT at 5 min, there were no significant differences between the groups. As expected based on findings presented earlier, at the time of syncope, catecholamine levels in HUT-positive patients were higher than baseline levels and higher than in HUT-negative patients. Similarly, cAMP levels increased at syncope and were higher than in non-syncopal patients at the end of the HUT (607 ± 460 vs. 328 ± 297 nmol/ml).

In summary, while the investigators have raised the possibility that c-AMP may be relevant to the pathophysiology of an evolving VVS, it is reasonable at this point to conclude that c-AMP levels are primarily driven by the catecholamine changes summarized earlier.

#### Endogenous Opioids

Endogenous opioids have been considered as agents potentially triggering or otherwise contributing to VVS (13, 46). Several observations favor this possibility: (1) the high concentration of neural opioids in the mid-brain cardiovascular centers, (2) previous findings indicating that naloxone diminishes baroreceptor sensitivity, (3) opioid agonists have been shown to trigger a vasovagal-like response to experimental hemorrhage, and (4) beta-endorphins have been shown to be increased in association with VVS. Further, in an initial study, Wallbridge et al. (13) noted beta-endorphin increase prior to the faint.

Perez-Paredes et al. (46) evaluated the role of endogenous opioids in neurally-mediated syncope. Head-up tilt test was performed on 35 patients with syncope of unknown origin. Subjects with a positive drug-free HUT showed a larger rise in plasma beta-endorphin concentrations at time of syncope (baseline 13.7+/-8.0 vs. syncope 41.4 ± 26.4 pmol/l; *P* < 0.01). On the other hand, patients with a positive isoproterenol-test showed no rise in plasma beta-endorphin levels as was also the case for subjects with negative HUT tests. However, intravenous naloxone at a dose of 0.02 mg/kg was not superior to placebo for preventing positive responses to baseline HUT.

In summary, the role that opioids may play in VVS is uncertain. However, given the lack of utility of opioid agonists for preventing VVS, it is unlikely that the opioid contribution is crucial to VVS pathophysiology.

#### Plasma Renin Activity and Angiotensin II

Jardine et al. (14) provided the first assessment of renin and angiotensin II in HUT-induced VVS. Findings show that renin increased substantially in control non-fainters, but only modestly in fainters. Similarly, angiotensin II increased somewhat in both groups, but remained higher in non-fainting controls. Gajak et al. (47) examined plasma renin activity (PRA) and found that it increased throughout the HUT procedure and was ~2.5-fold greater than baseline at the time of syncope. However, there were no comparative control data offered. In this regard, Vanderheyden et al. (15) observed that patients with cardioinhibitory syncope exhibited blunted activation of the renin-angiotensin-aldosterone axis at syncope. Conversely, the renin-angiotensin-aldosterone axis is activated in patients with vasodepressor syncope and in patients with a negative result of head-up tilt test.

Angiotensin II antibodies and antiadrenergic antibodies have been postulated to play a role in postural orthostatic tachycardia syndrome. A similar finding might reasonably be anticipated in individuals susceptible to VVS. However, Yu et al. did not find this to be the case (48).

In brief, the role of PRA/angiotensin II in VVS pathophysiology remains unclear. Additional controlled observations are needed.

#### Serotonin

Serotonin (5-hydroxytryptamine) is derived from neural and gastrointestinal sites, with subsequent storage predominantly in platelets, and has a number of effects on the central nervous system as well as the endocrine and cardiovascular systems. Matzen et al. (49) raised the possibility that inasmuch as serotonin has been associated with BP regulation, and is well-represented in areas of the brain pertinent to VVS, it may be a contributor to VVS by virtue of central-mediated anti-sympathetic effects. In an initial study these authors found that serotonergic stimulation using the re-uptake inhibitor clomipramine (with prolactin and cortisol as biomarkers) resulted in a greater effect in patients with presumed VVS than in control subjects (49). Later, the same group reported the effects of clomipramine during HUT (50). In this report, all individuals had been previously tested using 60 degrees HUT for 30 min, and if negative, with isoproterenol provocation. Baseline HUT was positive in 23/55 cases (53%) patients and none of 22 controls. However, after clomipramine, HUT was positive in 80% of patients, but only one control subject. The authors concluded, based on these 2 studies, that enhanced serotonin responsiveness with clomipramine leading to a presumed (but not proven) greater sympatholytic effect, supports a mechanistic role for serotonin in the central pathways leading to VVS initiation.

Unfortunately, interpretation of the clomipramine observation is clouded by the fact that the drug impacts many other receptor sites (e.g., histamine H1, norepinephrine re-uptake [NET], and muscarinic sites). Further, clomipramine metabolites exhibit a greater blocking affinity for NET than for the serotonin re-uptake site. Consequently, studies with clomipramine can only be taken as suggestive of serotonin contribution to VVS. Furthermore, others have provided findings that seem to dispute an active role of serotonin in VVS. For example, Matzen et al. (49) found that while methylsergide (a serotonin receptor blocker) did alter a number of neurohumoral responses (e.g., NE, PRA) during HUT, it did not alter hypotensive responses. Similarly, Alboni et al. (51) did not find a substantial change in plasma or platelet serotonin levels during HUT-induced VVS. Consequently, the available literature does not support a crucial place for serotonin in the initiation of VVS.

In summary, a putative role for serotonin in VVS remains debatable. For the most part selective serotonin reuptake inhibitors (SSRIs) have not proved effective clinically for preventing VVS. Nevertheless, further evaluation of the serotoninergic pathways with more specific blockers is warranted.

## Possible Implications of Neurohumoral Agents in VVS Pathophysiology

Humans are believed to be the principal if not the only species exhibiting VVS. However, VVS susceptibility does seem to differ among individuals, and also varies over time within affected persons. Thus, while 20–30% of humans report having had a presumed VVS event in their lifetime, only a much smaller proportion exhibit multiple episodes (1–4, 8). Further, even among patients in whom VVS is known to have occurred, repetitive exposure to the same stimulus may not consistently trigger an episode. The latter is exemplified, for example, by lack of reproducibility of VVS induction during HUT (52). Consequently, while inter-individual genetic issues may account for some differences among individuals, they cannot account for intra-individual variations in susceptibility. In this regard, alterations in factors such as hydration, environmental exposure (e.g., temperature, stress, etc.) and neurohumoral status may be relevant.

Several hypotheses have been put forward in an attempt to understand VVS pathophysiology (see Ref #3 for summary). However, none provide a comprehensive explanation for how a VVS episode is first triggered, then evolves, and ultimately self-terminates. The understanding that does exist is principally based on observations obtained in VVS induced by upright posture or by LBNP (as opposed for example to emotional or pain triggers).

### Initial VVS Stage

It is generally agreed that in orthostatic-triggered VVS the initial and perhaps crucial event is diminution of venous return to the heart with associated fall of stroke volume (SV) and cardiac output (CO). The venous reservoir below the diaphragm is very large and compliant; if dilated by neural and/or humoral agents, blood pooling may occur which is far in excess of the usual volume associated with movement to upright posture. The dependent sites which are particularly likely to pool blood are the thighs, buttocks, pelvic organs and very importantly the splanchnic bed.

Apart from the altered concentrations of circulating catecholamines (mainly Epi and NE) as VVS evolves, there is in addition a change in the Epi/NE ratio (see earlier discussion). Potentially, the greater the circulating Epi/NE ratio, such as has been documented in an evolving VVS event (see above), the greater the potential dependent pooling.

The functional impact of a greater Epi/NE ratio during an evolving faint is not proven but as pointed out by Goldstein et al. (6) it appears that activation of the sympathetic neural and adrenal sympathetic system do not necessarily operate in concert (“sympatho-adrenal imbalance”). Thus, while Epi is usually a predominant vasoconstrictor (alpha-adrenergic action) in the cutaneous circulation (which likely accounts for the pallor associated with VVS), the constrictor effect is not a universal Epi action. In the case of VVS, Epi concentrations in excess of NE may be expected to dilate certain skeletal muscle beds and thereby contribute to venous pooling. Further, at high concentrations Epi beta-adrenergic effects may be vasodilatory in both the splanchnic and hepatic circulation. The latter action may in part account for the abdominal sensation (often perceived as “nausea” or abdominal fullness) that often accompanies VVS.

A substantial increase in peripheral vasoconstrictor activity (e.g., exogenous NE infusion) may ameliorate or terminate the evolving VVS event in the initial stage of VVS, but with a higher than usual Epi/NE ratios, reversal of the evolving hemodynamic crisis may not occur unless the patient voluntarily assumes a gravitationally neutral posture.

Several reports note that the catecholamine changes begin at a time during HUT when patients are still hemodynamically stable, suggesting a contribution to the initiation of the event (10, 14, 53). Further, the increase is greater in subjects who go on to faint, than in those who do not. Moreover, among individuals who go on to faint, the greater the Epi increase and the higher the Epi/NE ratio early upon assuming upright posture, the greater the VVS susceptibility as measured by “time to syncope” (19, 22).

Despite the plausible role played by Epi and Epi/NE ratio in triggering VVS, there is also strong evidence pointing away from these agents being causative. For instance, Calkins et al. did not find Epi to be useful for triggering VVS during tilt-testing (3, 54). More importantly though, while a number of small, single-center observational studies have suggested that beta-adrenergic blockade may be beneficial prophylaxis in VVS patients, the broader clinical experience is that beta-adrenergic blockers typically do not provide protection against VVS recurrences in most patients (1, 2, 8, 23). In this regard, Sheldon et al. (23) observed only a trend toward utility of beta-adrenergic blockers in older (>42 years) but not younger VVS susceptible patients. On the other hand, as has been suggested by others (55, 56), older patients appear to release less epinephrine than do younger individuals. Perhaps, it may be possible to achieve adequate blockade with tolerable beta-blocker doses in older individuals, while that might not be achievable in younger patients.

Reduction of venous return to the heart diminishes SV and CO. In otherwise healthy individuals, this scenario not unexpectedly triggers an increase in heart rate (HR). The resulting presumably compensatory positive chronotropic drive, is most likely initiated by signals derived initially from low pressure cardiac receptors, as well as potentially somewhat later by higher pressure arterial receptors; the result is parasympathetic “withdrawal” and sympathetic neural activation. The role of neurohumoral agents at this stage is uncertain. However, the impressive rise of epinephrine levels early during the evolving faint is likely important.

Ultimately, diminished venous return prevents an increased heart rate from providing adequate hemodynamic support. Consequently, given the falling CO despite the attempted HR compensation, one might also expect total peripheral resistance (TPR) to rise in conjunction as sympathetic neural activation attempts (at least in healthy circulations) to maintain blood pressure (BP). However, while HR rises, TPR change is less clear-cut in VVS. Perhaps the explanation in part relates to the altered circulating Epi/NE ratio with constriction in some beds and vasodilation in others as noted earlier. However, as time goes by, other hormonal contributors may become important, particularly vasopressin (AVP).

### Second Phase

As the VVS episode evolves, usually over several minutes, a modest but important downward BP trend has been well-described; this occurs typically at a time when HR is still higher than baseline (57–59). Why the heart rate is not driven to very high levels at this time is has not yet been fully explained. However, one may speculate that a balance is developing between the former Epi predominance, and resurgence of parasympathetic activity.

Finally, in this stage of the evolving faint, several factors may undermine any attempt by the vascular system to effect a sufficient increase of TPR to compensate for the low CO. In essence, as alluded to previously, sympathetic constrictor neural effects may be counteracted in critical portions of the circulation by the adverse Epi/NE ratio. However, in addition, to the disadvantage of the circulation, other humoral agents may tilt the balance to a dilator direction (particularly, adrenomedullin and ANP being dilators) in conjunction with the diminished or ineffective humoral constrictor effects of agents such as ET-1 and AVP as discussed above.

### Final Phase

As syncope approaches, there is a marked and usually abrupt drop in BP (55). HR at this stage (even with rapid pacing) cannot prevent hemodynamic collapse; in fact, a “relative” (vis-à-vis the magnitude of hypotension) or at times marked (asystole for often lengthy periods) bradycardia ensues. This reversal of HR trend (from relatively stable tachycardia to bradycardia) is often attributed to the action of ventricular mechanical receptors in the setting of a perceived “empty” ventricle (3). Whether the latter mechanism is valid or not, the HR slowing is well-known to be reversible with muscarinic blockade (i.e., atropine) (60) and thus reflects abrupt increase of parasympathetic tone. Nevertheless, it is rare that the BP fall is ameliorated by increasing HR. The moment at which a physiologically useful HR increment (whether by medication or pacing) can be introduced, is now past.

The basis for this apparent abrupt reversal of parasympathetic tone may be in part driven by humoral factors. As noted earlier, AVP increases markedly late in the VVS event; the trigger for this is uncertain but could be compensatory given AVP's combination of vasoconstriction and anti-diuretic actions. However, AVP in humans only exhibits vasoconstriction at high concentrations, and such high concentrations are likely reached only in the late stages of a VVS episode when its vasoconstrictive effect may be too late to be effective. More pertinent, however, vasopressin is also known to stimulate the baroreceptor system which diminishes sympathetic activity and on balance enhances parasympathetic influence (24, 32, 61). The net outcome might then not promote circulatory homeostasis, but in fact a contributor to hypotension.

Loss of consciousness usually leads to falling, with the individual assuming a horizontal position. The latter is a gravitationally neutral position that permits recovery of venous return, normalization of SV and ultimately of BP as sympathetic tone normalizes. Of note, a post-event overshoot of BP (as is seen in neurogenic orthostatic hypotension or with a normal Valsalva maneuver) is not a characteristic of VVS recovery. Consequently, while total peripheral resistance (TPR) increases toward normal after a VVS event, it does not seem to ever reach super-normal values such as commonly occurs with the more transient diminution of cardiac output and pulse pressure in the Valsalva maneuver. Nevertheless, the hemodynamic situation is precarious. If the patient is moved to an upright position, too soon, then a second faint may be triggered.

## Conclusions

A number of neurohormonal agents have been studied to ascertain both how they change during posture-induced vasovagal faints and whether they contribute to the phenomenon. Nonetheless, despite the important insights that have been gained, many elements of the puzzle remain unclear. In particular, why VVS susceptibility varies between individuals and over time within known fainters remains a mystery that is not readily accounted for by either genetic, neural or hormonal differences. At this time, although there is reasonable evidence implicating Epi, NE and AVP changes in certain aspects of VVS, whether they or other neurohormones contribute directly to the evolving faint is still debatable. Further, whether better understanding of neurohumoral effects would enhance VVS prevention remains uncertain. Additional study is needed.

## Author Contributions

DB developed the manuscript and developed the text and Figures. JD provided critical information related to neurohormones. DK reviewed and corrected the text and offered key material. WA provided criticism and key revision. SS revised the text and provided key material.

## Conflict of Interest

The authors declare that the research was conducted in the absence of any commercial or financial relationships that could be construed as a potential conflict of interest.
